# Clinical Feasibility and Familiarization Effects of Device Delay Mismatch Compensation in Bimodal CI/HA Users

**DOI:** 10.1177/23312165231171987

**Published:** 2023-05-17

**Authors:** Julian Angermeier, Werner Hemmert, Stefan Zirn

**Affiliations:** 1 Faculty of Electrical Engineering, Medical Engineering and Computer Sciences, Peter Osypka Institute of Medical Engineering, University of Applied Sciences Offenburg, Offenburg, Germany; 2 Bio-Inspired Information Processing, Munich Institute of Biomedical Engineering, 9184 Technical University of Munich, Garching, Germany

**Keywords:** bimodal hearing, sound localization, device delay mismatch

## Abstract

Subjects utilizing a cochlear implant (CI) in one ear and a hearing aid (HA) on the contralateral ear suffer from mismatches in stimulation timing due to different processing latencies of both devices. This device delay mismatch leads to a temporal mismatch in auditory nerve stimulation. Compensating for this auditory nerve stimulation mismatch by compensating for the device delay mismatch can significantly improve sound source localization accuracy. One CI manufacturer has already implemented the possibility of mismatch compensation in its current fitting software. This study investigated if this fitting parameter can be readily used in clinical settings and determined the effects of familiarization to a compensated device delay mismatch over a period of 3–4 weeks. Sound localization accuracy and speech understanding in noise were measured in eleven bimodal CI/HA users, with and without a compensation of the device delay mismatch. The results showed that sound localization bias improved to 0°, implying that the localization bias towards the CI was eliminated when the device delay mismatch was compensated. The RMS error was improved by 18% with this improvement not reaching statistical significance. The effects were acute and did not further improve after 3 weeks of familiarization. For the speech tests, spatial release from masking did not improve with a compensated mismatch. The results show that this fitting parameter can be readily used by clinicians to improve sound localization ability in bimodal users. Further, our findings suggest that subjects with poor sound localization ability benefit the most from the device delay mismatch compensation.

## Introduction

Sound localization is an important ability in everyday life. From localizing an approaching car to spatial unmasking in multi-talker environments, it helps humans to navigate in everyday environments. In bimodal listeners provided with a hearing aid (HA) in one ear and a cochlear implant (CI) in the contralateral ear, sound localization is significantly impaired compared to normal-hearing listeners (Dorman et al., 2018; Seeber et al., 2004; Veugen, Hendrikse, et al., 2016). One possible explanation of this poor performance relates to mismatches between the two stimulation modalities (Pieper et al., 2022). In bimodal listeners, the two modalities can be theoretically mismatched in three dimensions: level, frequency, and latency. In the domain of level, this mismatch has its origin in both devices usually being fitted independently, with little attention paid to the balancing of both devices in terms of perceived loudness. Further, the optimal strategy to balance loudness in bimodal listeners is subject of ongoing research (Francart et al., 2012; Veugen, Chalupper, et al., 2016). In the case of frequency mismatch, the limited insertion depth of the CI electrode makes it hard to stimulate the apex of the cochlea. Therefore, the most apical electrodes usually stimulate more basal regions than the same input would stimulate in the acoustic ear. This is due to the default setting for the frequency-to-electrode mapping often being used, irrespective of the insertion depth of the CI electrode. Such a mismatch between the two ears has been shown to reduce the binaural benefit such as ITD and ILD sensitivity and speaker separation in CI users with single-sided deafness (SSD), bilateral users of CIs and in normal-hearing listeners (Bernstein et al., 2018; Francart & Wouters, 2007; Hu & Dietz, 2015; Wess et al., 2017). However, Dirks et al. (2022) could not show improvements in sound localization or speech understanding in noise in SSD CI users after compensating for this frequency mismatch, stating the last mismatch to be discussed in this introduction as a possible reason. This last mismatch is in latency. In bimodal listeners and to some extent in SSD listeners, a systematic offset in stimulation timing is present. This offset originates from different processing latencies and stimulation pathways in the two devices, potentially leading to a delayed stimulation of the auditory nerve in the ear provided with the HA compared to the CI-aided ear in users (Zirn et al., 2015). This mismatch impairs sound localization and has been termed device delay mismatch (Zirn et al., 2019). All three of these dimensions of mismatch are thought to interact with each other. In this work, the effects of a device delay mismatch in bimodal listeners were investigated separately from the other potential mismatches. Previous studies reported a significant improvement in sound source localization accuracy when this device delay mismatch was reduced (Angermeier et al., 2021; Zirn et al., 2019), irrespective of mismatches in level or frequency. However, these studies utilized experimental signal processing to delay CI stimulation in the form of programmable, wearable delay lines (DL). These experimental setups were worn around the neck and had the shortcoming that effects of familiarization periods of several weeks could not be investigated. Long-term effects become especially interesting when results presented by Trapeau and Schönwiesner (2015) are considered. In their study, familiarization to a systematic delay of 625 µs on one ear was investigated in normal-hearing subjects over several days. Subjects showed decreased localization accuracy that was subject to familiarization over the course of one week, with the most significant effects of familiarization taking place after one day. However, familiarization was not able to completely compensate for the detrimental effect of this unilateral delay. It is also important to note, that the delays used in this study were much smaller than the mismatches observed in bimodal listeners. Therefore, it is not clear, whether the mechanisms observed in this study could also benefit bimodal users.

The CI manufacturer MED-EL offers the possibility to delay CI stimulation in their fitting software in the latest generation of speech processors. Further, they supply clinicians with a list of HA delays from the HA manufacturers. Since HA latencies can vary slightly, even depending on the pre-processing settings, as shown by Alexander (2016), it is unclear whether these standard HA delays provided by the manufacturers provide full improvement in sound source localization. Thus, the effects of using standard HA latencies that vary slightly from the measured HA latencies on sound localization were investigated in the presented study.

Another topic that has so far only been investigated in normal-hearing listeners is speech understanding and especially the improvement in speech reception thresholds (SRTs) when the masker and the target are spatially separated compared to the situation when target and masker come from the same direction, the so-called spatial release from masking (SRM); for an overview of SRM, see Litovsky (2012) . In normal-hearing listeners, an increased device delay mismatch has been shown to have a significant influence on ITD-based SRM but not on ILD-based SRM (Angermeier et al., 2022). Other studies only investigated the influence of a device delay mismatch in hearing impaired listeners on SRTs when speech and noise were presented from the same loudspeaker in front of the subjects and could not report any effect of device delay mismatch in SSD subjects (Seebacher et al., 2019). This is not too surprising, however, since speech understanding is not affected by binaural cues when both masker and target are collocated. It is currently believed, that SRM in bimodal subjects is mainly moderated by one ear having a better signal-to-noise ratio (SNR) and thus being driven by monaural factors that should in theory not be affected by a device delay mismatch (Dieudonné & Francart, 2020; Dirks et al., 2019; Williges et al., 2019). However, an influence of a device delay mismatch on these findings has not been investigated systematically so far, leaving the question whether this missing access to binaural mechanisms in SRM is due to impaired binaural processing in subjects experiencing such a mismatch.

The primary aim of this study was to find out if the compensation of the device delay mismatch can be performed within the MAESTRO 9 software based on the manufacturers’ HA delay values. This is especially interesting for CI audiologists who do not have access to measurement devices to determine the HA delays themselves. Secondary aims of this study were the investigation of the effects of longer familiarization periods on the effects demonstrated in earlier studies and the effects of a device delay mismatch compensation on speech understanding in noise in bimodal subjects.

## Methods

### Subjects

Eleven bimodal subjects participated in this study (8 male/3 female), with a mean age of 58.7 years (min: 33; max: 73). Inclusion criteria for this study were everyday use of both CI and HA and a bimodal experience of at least 6 months. Details about the subjects are listed in Table 1.

**Table 1.** Data of all Bimodal Subjects (CI = Cochlear Implant; HA = Hearing Aid).

Subject	Age	Aetiology	Implanted side	CI (implant/electrode)	CI experience [years]	HA experience [years]	CI coding strategy
Bim201	56	Progressive	Left	CONCERTO/Flex 28	1.5	6.5	FS4-p
Bim202	72	Sudden hearing loss	Right	CONCERTO/FlexSoft	9	30	FS4
Bim203	62	Blast trauma	Right	SYNCHRONY/Flex 28	2.5	6.5	FS4
Bim204	60	Sudden hearing loss	Left	SYNCHRONY/Flex 28	2.5	9.5	FS4-p
Bim205	65	Sudden hearing loss	Right	SYNCHRONY/Flex 28	6	4.5	FS4
Bim206	67	Unknown	Right	SYNCHRONY/Flex 28	3	3.5	FS4
Bim209	48	Unknown	Left	SYNCHRONY/Flex 28	6	19	FS4
Bim210	43	Progressive	Right	SYNCHRONY/Flex 28	6	8	FS4
Bim211	33	Progressive	Left	SYNCHRONY/Flex 28	2.5	15	FS4
Bim212	73	Progressive	Right	SYNCHRONY/Flex 24	2.5	51	FS4
Bim213	67	Acute hearing loss	Left	SONATA/Standard	13	41	FS4

Subjects Bim201, Bim202, Bim203, Bim204, Bim205, Bim206 and Bim209 had already participated in an earlier study (Angermeier et al., 2021). All subjects had complete insertions of their CI electrode except for subject Bim206, who had an incomplete insertion of the electrode array and thus did not use electrodes 11 and 12. Bim212 had electrode 12 deactivated due to poor sound quality. Subject Bim209 had electrode 5 deactivated due to excessive noise from this electrode. Finally, subject Bim213 had electrode 4 deactivated because of subjective disturbance of sound quality. All testing was conducted in accordance with the Code of Ethics of the World Medical Association (Declaration of Helsinki) for experiments involving humans. Approval by the Technical University of Munich ethics committee was obtained (340/19). All subjects gave written informed consent for each individual study date and were financially compensated for their travel expenses and time.

**Figure 1.**
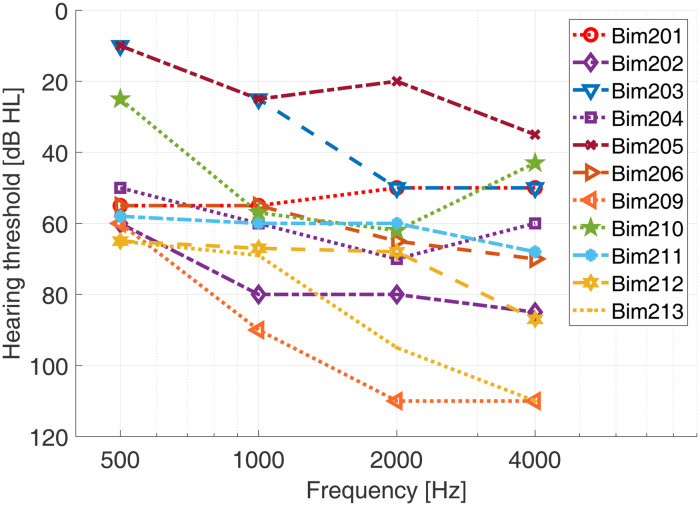
Hearing thresholds of the 11 subjects for the ear provided with a HA.

For the ear provided with the HA, the subjects’ hearing loss ranged from mild to severe hearing loss (Figure 1). For the ear provided with the CI none of the subjects had residual hearing at the acoustic frequencies of 0.5, 1, 2, and 4 kHz.

### Experimental Procedure

The study consisted of two study dates with a testing duration of 2–3 hours each. All study dates took place in the NeuroAkustik laboratory of the University of Applied Sciences Offenburg. Between each study date, a familiarization period of 3–4 weeks was administered to investigate the effect of longer familiarization periods compared to previous studies (Angermeier et al., 2021; Zirn et al., 2019). We used an A-B-B-A test design to measure the effects of a mismatch compensation as in a previous study (Table 2). In condition A, there was no compensation of the device delay mismatch and the everyday baseline performance of the subjects was measured. In the first B condition, we performed measurements acutely after compensation of the device delay mismatch. The second B condition was performed on the following study date, thus including familiarization period of 3–4 weeks to the adjusted delay. Finally, the second A condition was measured acutely after switching off the device delay mismatch compensation. With this study design, acute improvements or deteriorations can be measured by comparing the first A and B condition and the second B and A condition. Improvements due to familiarization can be investigated by comparison of the first and second B conditions. With the comparison of the first A condition versus the second A condition, learning effects over the course of the study and effects of familiarization to a device delay mismatch can be investigated.

**Table 2.** Overview of the Experimental Procedure for Both Study Dates (CI = Cochlear Implant; HA = Hearing Aid).

Study date	Condition	CI/HA directionality	Programmed CI Delay	Tests performed
1^st^	A	Natural/default settings	None (=1.5 ms)	Sound localization Speech in Noise
1^st^	B	Natural/default settings	Manufacturer delay for subjects HA/HA delay measured (only used for sound localization)	Sound localization Speech in Noise
**3–4 weeks familiarization**				
2^nd^	B	Natural/default settings	Manufacturer delay for subjects HA	Sound localization Speech in Noise
2^nd^	A	Natural/default settings	None ( = 1.5 ms)	Sound localization Speech in Noise

### CI Fitting

On the first study date, all subjects received a MED-EL SONNET 2 speech processor (MED-EL, Innsbruck, Austria) as an experimental device for the entire duration of the study. This processor in combination with the fitting software MAESTRO 9 has implemented an adjustable programmable delay of the CI stimulation of up to 20 ms with a 0.1-ms resolution within its fitting software. This was true for all subjects except subject Bim202 who already owned a SONNET 2 but did not use the adjustable delay before the study. In MAESTRO 9, a delay of the CI stimulation can be activated by adding a HA on the contralateral side to the CI and typing in a specific HA delay by which the CI stimulation should be delayed. After initial fitting, we asked the subjects to adjust the loudness of the study processor to their own processor via their remote control. This was performed in the lab while talking to the subjects and having them adjust the loudness of the perceived speech to match the loudness of their own processor. In each fitting session, subjects were asked to adjust the perceived loudness so that it matched with the loudness of the prior fitting. The subjects used their everyday map with their desired pre-processing enabled (e.g., wind reduction, etc.). In cases where the subjects had adaptive beamforming enabled, the directionality was changed to “natural” within the fitting software, mimicking the directionality of a human pinna. For the initial test, the adjustable delay of the CI speech processor was set to the minimum value of 1.5 ms. For the testing with the compensated device delay mismatch, delays were used for the subjects’ HA which can be found online (a corresponding link appears in the fitting software provided by MED-EL: https://www.medel.pro/online-resources/timing-settings-for-hearing-aids). No further alterations to the CI fitting were performed. After the second B condition, the CI delay was acutely reset to minimal value of 1.5 ms.

### HA Fitting

All subjects used their own HA throughout the study. On the first study date, HA delays were measured for each subject with the method described in Angermeier et al. (2021). No changes were made to the subjects HA directionality settings. An overview of the subjects’ HAs with their respective delays given by the manufacturer and the delays measured in the lab can be found in Table 3. HA delays were defined as frequency independent since the MAESTRO 9 software only allows for a frequency independent delay for the CI stimulation. The HA delay given by the manufacturers were on average 5.2 ± 2.1 ms with a maximum delay of 8.1 ms for Bim213 and a minimum delay of 2 ms for Bim203 and Bim205. The HA delays measured in the laboratory via a self-designed measurement setup averaged at 5.2 ± 2.4 ms with a maximum delay of 7.8 ms for Bim202 and a minimum delay of 2.8 ms for subjects Bim203, Bim205 and Bim206. The mean absolute difference between manufacturer given delay and measured HA delay was 0.68 ms with a maximum difference of 2.2 ms for Bim210. These differences are probably due to differences in measurement setups to determine the HA delays.

**Table 3.** Hearing Aid Types and Processing Delays Given by Manufacturers and Measured During the Study.

Subject	HA type	HA processing delay provided by the manufacturer [ms]	HA processing delay measured [ms]	Absolute difference [ms]
Bim201	ReSound LiNX2 LS9	5	5	0
Bim202	Oticon Xceed 2UP	8	7.8	0.2
Bim203	Widex ENJOY50 FM	2	2.8	0.8
Bim204	Oticon Ruby 2 PP	8	7.5	0.5
Bim205	Widex Daily50 D-FA	2	2.8	0.8
Bim206	Widex Beyond 330 B3-F2	2.9	2.8	0.1
Bim209	Phonak Naida Q90 UP	6.1	7.2	1.1
Bim210	Audio Service SUN	6.2	4	2.2
Bim211	Phonak Audeo V90-13 RIC	6.1	6.3	0.2
Bim212	Widex Unique Fs 330	2.9	3.5	0.6
Bim213	Phonak Naida B90 UP	8.1	7.1	1

### Test Environment

All testing was conducted in an audiometric booth (IAC Acoustics, Niederkrüchten, Germany). Subjects were seated in the center of a loudspeaker circle consisting of twelve loudspeakers (type Genelec 8030C, Genelec Oy, Iisalmi, Finland) with an angular spacing of 30° and a diameter of 2 m. The loudspeakers were located 1.15 m above the floor, approximately aligning the center of the loudspeaker membranes with the ears of the seated subjects. Loudspeakers were labeled clockwise with numbers between 1 and 12 with speaker number 1 representing −90° azimuth. All stimuli were presented via a RME Fireface 802 soundcard (Audio AG, Haimhausen, Germany) which was connected to a measurement PC outside the audiometric booth. Answers were captured using a tablet computer and sent via Bluetooth to a PC outside the audiometric booth for processing in MATLAB (The MathWorks Inc., Natick, MA, USA).

### Sound Localization

Sound localization accuracy was measured in the frontal horizontal hemisphere using the seven loudspeakers between −90° and 90° azimuth with an angular spacing of 30° (loudspeaker 1 to loudspeaker 7). Stimuli consisted of five white noise bursts (125 Hz to 20 kHz). Each burst had a duration of 70 ms with 3 ms Gaussian-shaped slopes and pauses of 30 ms between each burst as described by Seeber et al. (2004) and as used in a previous study (Angermeier et al., 2021). Spectral roving with two different spectral characteristics (stimuli were filtered with the left- and right-ear HRTF recorded at a source position of 90° azimuth) and level roving (60, 65, and 70 dB) was applied to the stimuli in each trial to minimize the usage of monaural cues according to Van de Heyning et al. (2016). Stimulus generation was performed in MATLAB. Each localization test consisted of a total of 84 stimuli, presenting each combination of spectrum, level, and speaker position twice (2 × 2 × 3 × 7) in random order. Subjects had a visual representation of the seven loudspeakers with their respective speaker numbers in the frontal horizontal hemisphere on the tablet computer and responded to stimuli by clicking on the speaker that they assumed the stimulus was originating from. During testing, no feedback was given to the participants whether their responses were correct or wrong. Prior to the localization tests participants completed a training run in which feedback of the correct source position was given via the tablet after subjects submitted their answers. Subjects were instructed to face the loudspeaker with the number 4 which was at 0° azimuth. The chance level for the RMS error in this test setup was 84.7 ± 4°. For more details about the localization testing see Angermeier et al. (2021). Subjects performed one localization test for each delay condition. Additionally in the first B condition, acute localization tests with the manufacturer given HA delay and the measured HA delay were conducted to investigate differences between both delay values on sound source localization accuracy acutely.

### Speech Tests

To measure the effect of a compensated device delay mismatch on speech perception, the Oldenburg sentence test (OlSa) was used (Wagener et al., 1999). The OlSa is a German matrix sentence test with each sentence being made up of five words in the following manner: Name—verb—number—adjective—object. Sentences of the OlSa speech material are semi-nonsensical to avoid effects of guessing words based on the sentence's context. SRTs were measured, which is the SNR between speech signal and masking noise at which 50% of the speech material is understood correctly. Olnoise (i.e., the standard masking noise for the OlSa) was used as a masker, generated from the speech material overlapped 30 times with random temporal shifts creating a broadband noise masker with the same long-term average speech spectrum as the speech material. Each test list consisted of 20 test sentences. Subjects gave their answers via a tablet computer displaying the word material of the OlSa. Subjects were allowed to guess or to leave specific words blank if they did not hear the word. All subjects completed a training run to get used to the test and word material on each study date with no feedback given. To assess bimodal performance, we measured the effect of SRM, which is the difference in SRTs between speech signal and noise being spatially collocated versus when speech and noise being spatially separated. To measure SRM, SRTs were measured with speech and noise being spatially collocated at 0° azimuth (S_0_N_0_) and with the speech coming from 0° azimuth and the noise from either 90° or −90° azimuth, dependent whether subjects had their HA on the right or left ear (S_0_N_HA_). This spatial condition was chosen to assess whether the anticipated better-ear listening on the CI side due to the better SNR on the CI side would be affected by the CI delay. Test and retest were measured for both spatial configurations yielding a total of four test lists per condition (2 tests × 2 spatial configurations). Within these four test lists conditions were randomized and subjects were asked if they preferred a short break after completion of each list. SRM was calculated as the difference between the mean SRTs between test and retest measured at S_0_N_0_ and S_0_N_HA_.

### Data Analysis and Statistical Evaluation

To assess sound source localization accuracy, two metrics were calculated according to Yost et al. (2013). The root-mean-square (RMS) error, being a measurement of the precision of the subjects’ judgments and the signed bias, representing the systematic error or trueness of the subjects’ localization judgments. As a signed term the signed bias can either indicate a bias to the left, when negative or a bias to the right, when positive. Since this bias is believed to be dependent on the stimulation modality in bimodal subjects, signed bias data were normalized. This was done by inverting the measured signed bias of subjects wearing the CI on their left ear. Following, a positive signed bias always represents a systematic error towards the CI of the subjects. For a detailed description of this calculation, see Angermeier et al. (2021). Shapiro–Wilk tests were performed on all datasets to test for normality. Due to the consistent non-normal distribution of datapoints, non-parametric Friedman tests were used to test for differences between different conditions. In the case of significant outcomes, post hoc pairwise testing was applied via Wilcoxon signed-rank tests. Bonferroni–Holm corrections were applied to the resulting alpha levels to correct for multiple comparisons. For comparisons, where pairwise testing was not applicable (e.g., regression between HA delay and performance), linear regression was performed. These regressions, however, can only be seen as exploratory since the statistical power is not sufficient to draw strong conclusions with the size of our cohort. When comparing performance level and performance improvements, Oldhams method was used to avoid mathematical coupling (Chiolero et al., 2013; Oldham, 1962). An alpha level of 0.05 was used to determine statistical significance. We performed all statistical testing in MATLAB (Version R2019b).

## Results

### Sound Localization

#### Overall Sound Localization Accuracy

**Figure 2.**
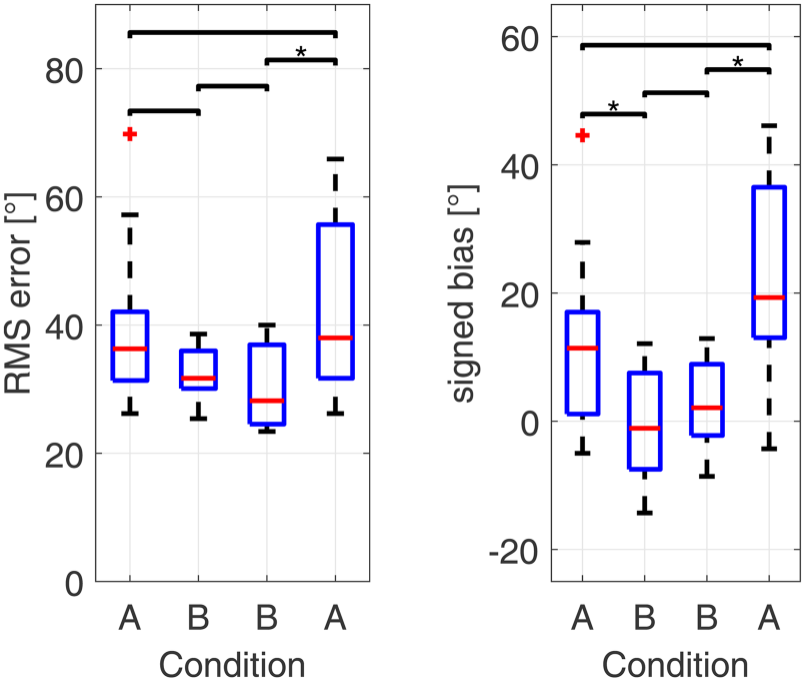
RMS error and signed bias between conditions as boxplots (red line: median; box: 1^st^ to 3^rd^ quartile; whiskers: minimum and maximum without outliers; outliers in red). Statistically significant differences denoted by *p < .05, **p < .01 and ***p < .001.

Sound localization accuracy in terms of RMS error and signed bias can be found in Figure 2. Friedman tests revealed a significant influence of condition for RMS error (*χ*^2^(3) = 13.69, *p* = .003) and for the signed bias (*χ*^2^(3) = 13.36, *p* = .004).

Post hoc pairwise Wilcoxon signed-rank tests revealed a significant improvement in sound localization accuracy between the first A and B condition in signed bias (*p* = .02) but not in RMS error indicating a significant improvement in the trueness of the localization judgments due to acute compensation of the device delay mismatch. After three weeks of familiarization no further significant difference was found in RMS error and signed bias when comparing the first and second B condition. When the CI delay was acutely set to its initial value of 1.5 ms, a significant deterioration could be seen in RMS error (*p* = .02) and signed bias (*p* = .02). Between the first and second A condition the RMS error did not change significantly. For the signed bias Wilcoxon signed-rank tests revealed a significant difference from a zero median for the first (*p* = .04) and second A condition (*p* = .009), and no significant difference from zero in the first (*p* = .8) and second (*p* = .48) B condition. Subject specific results for RMS error and signed bias can be found in the Supplemental Materials.

#### Effects of Delay Compensation Method

We investigated the effect of the delay compensation method acutely within the first B testing. A significant difference between delaying the CI stimulation by the manufacturer given delay and the delay measured in the lab could be found for RMS error (*p* = .01) with a median RMS of 31.7° (Q1: 30°; Q3: 36°) for the manufacturer delay and 34.9° (Q1: 31.4°; Q3: 40°) for the measured HA delay. However, with a mean difference of 2.8° in RMS error this significant difference has little relevance to the effectiveness of this fitting strategy. For the signed bias, no significant difference between the two delays could be found (*p* = .06). Thus, the delay chosen to compensate for the device delay mismatch did only significantly affect the precision of the subject's localization judgments but not the trueness, which is probably due to the relatively small difference between both delays (0.7 ± 0.6 ms).

#### Effects of Initial Localization Performance and HA Delay

**Figure 3.**
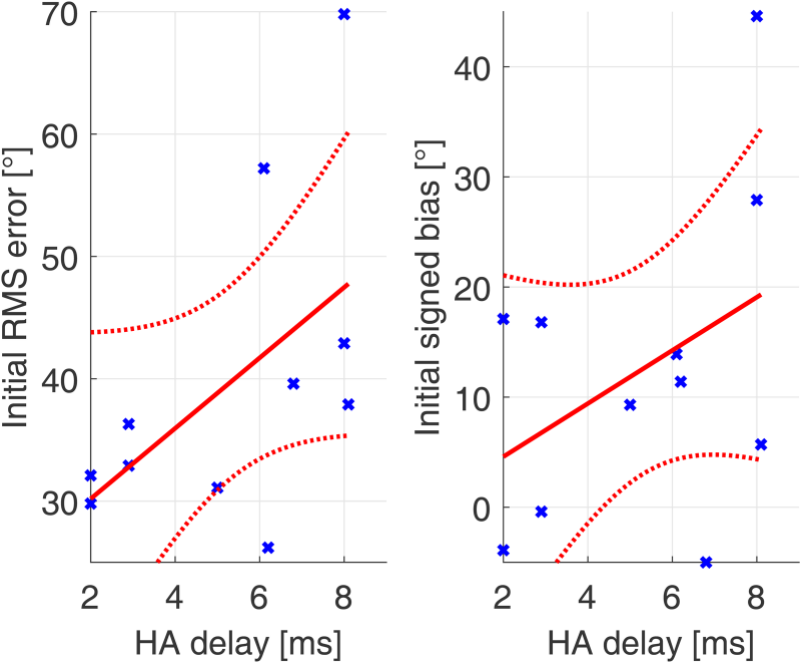
Linear regression between initial performance in RMS error and signed bias and HA delay. Data in blue, regression in red (dotted lines represent the confidence bounds of the linear model).

Investigating the question whether the HA delay and thus the magnitude of the device delay mismatch played a role in initial sound source localization performance, linear regression was performed between HA delay and the RMS error and signed bias in the first A condition (Figure 3).

No significant correlation could be found between HA delay and initial performance in RMS error (*r* = .54; *p* = .09) or signed bias (*r* = .41; *p* = .22). This means that the initial performance after familiarization to a device delay mismatch was not significantly correlated to the magnitude of this mismatch in our cohort.

#### Effects of Compensated Delay Magnitude

**Figure 4.**
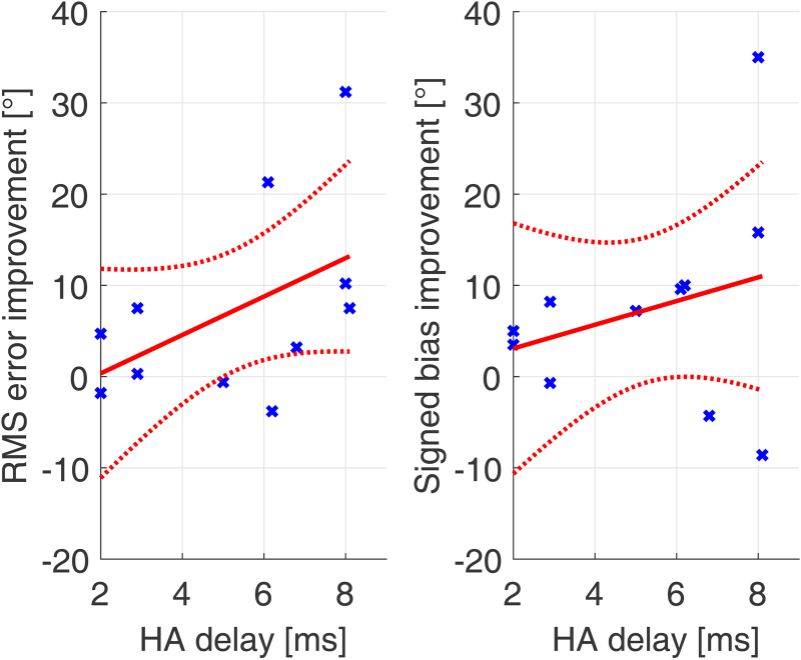
Linear regression between performance improvement due to device delay mismatch compensation in RMS error and signed bias and HA delay. Data in blue, regression in red (dotted lines represent the confidence bounds of the linear model).

To assess the effects of the magnitude of the corrected delay to the increase in performance in bimodal subjects, linear regression between HA delay and acute performance increase between the first A and B conditions were performed. The results can be found in Figure 4.

The improvement in signed bias was calculated as the decrease in distance to a signed bias of 0° since this is the optimal case. This was calculated as the difference to 0° in absolute signed bias of the first A and B condition. No significant correlation between HA delay and improvement in sound source localization in RMS error (*r* = .5; *p* = .13) or signed bias (*r* = .28; *p* = .41) could be found. However, a trend in the datasets can be observed.

#### Effects of Mean Performance

**Figure 5.**
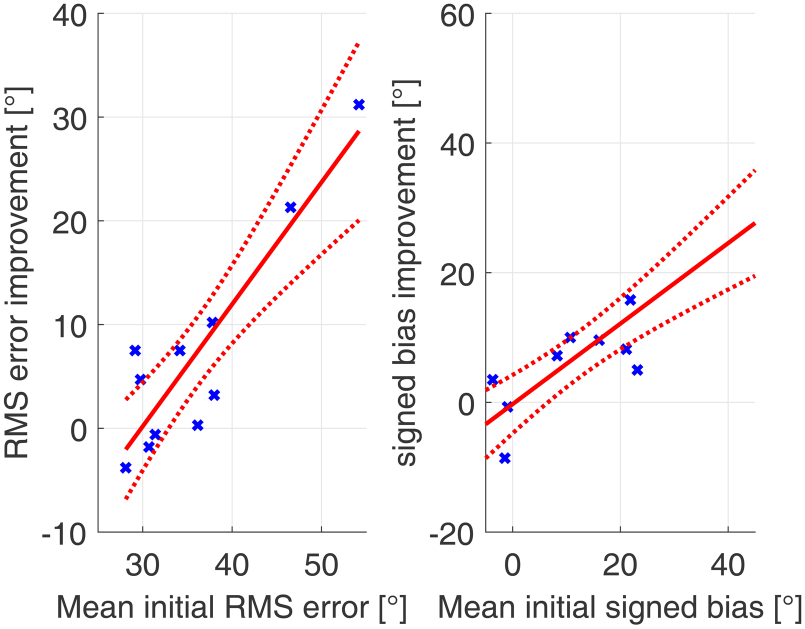
Linear regression between mean performance and acute improvement for RMS error and signed bias. Data in blue, regression in red (dotted lines represent the confidence bounds of the linear model).

Figure 5 shows the linear regression between the mean localization performance with and without the device delay compensation (mean of first A and first B condition) and improvement after acutely compensating the device delay mismatch. For the RMS error, mean performance and improvement of RMS error by device delay mismatch compensation correlated highly significantly (*r* = .9, *p* < .001). Also, for the signed bias, the correlation was highly significant (*r* = .9; *p* < .001).

It must be noted that the improvement in signed bias was calculated as the decrease in distance to a signed bias of 0° since this is the optimal case. This was calculated as the difference in absolute signed bias of the first A and B condition. These correlations revealed that subjects performing worse overall benefitted more from the compensation of the device delay mismatch than the subjects with overall better sound source localization accuracy without device delay mismatch compensation. The regression also shows, that in subjects with already good mean performance in terms of RMS error, the reduction of the device delay mismatch leads to a slightly negative RMS error improvement.

#### Effects of Acute Desynchronization of CI and HA Stimulation Versus HA Delay

Since a significant deterioration for the RMS error and signed bias between the second B and second A condition could be observed, linear regression between the HA delay and the deterioration between the second B and second A conditions was performed. This addressed the question whether this acute decline in sound source localization accuracy was related to the magnitude of device delay mismatch. This could not be shown before, when subjects were already familiarized to the device delay mismatch.

**Figure 6.**
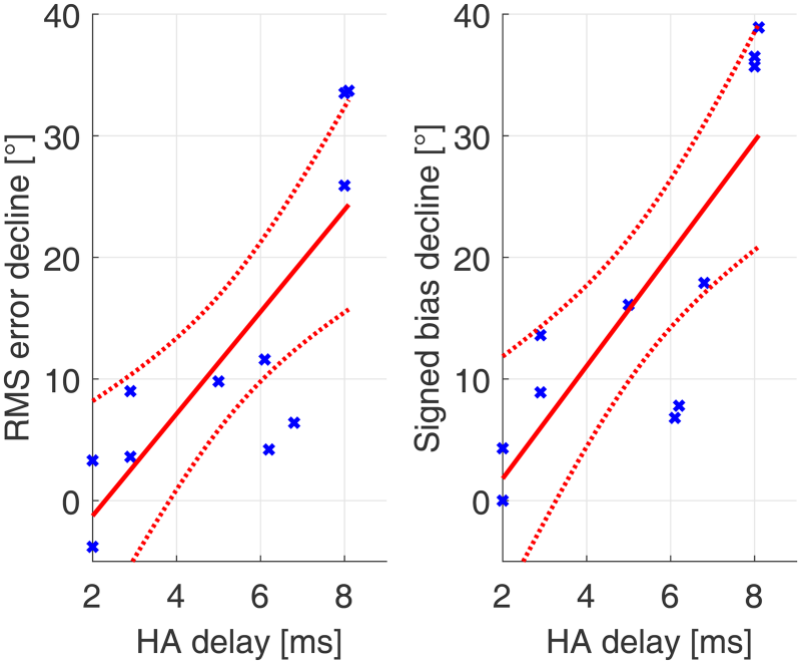
Linear regression between HA delay and acute performance decline for RMS error and signed bias. Data in blue, regression in red (dotted lines represent the confidence bounds of the linear model).

Figure 6 shows the linear regression between HA delay and performance decline for RMS error and signed bias. Note that for this figure, a positive decline means a higher decline between B and A.

There was a significant correlation for RMS error decline and HA delay (*r* = .8; *p* = .003) and between signed bias decline and HA delay (*r* = .81; *p* = .002).

### Speech Tests

#### Spatial Release from Masking

**Figure 7.**
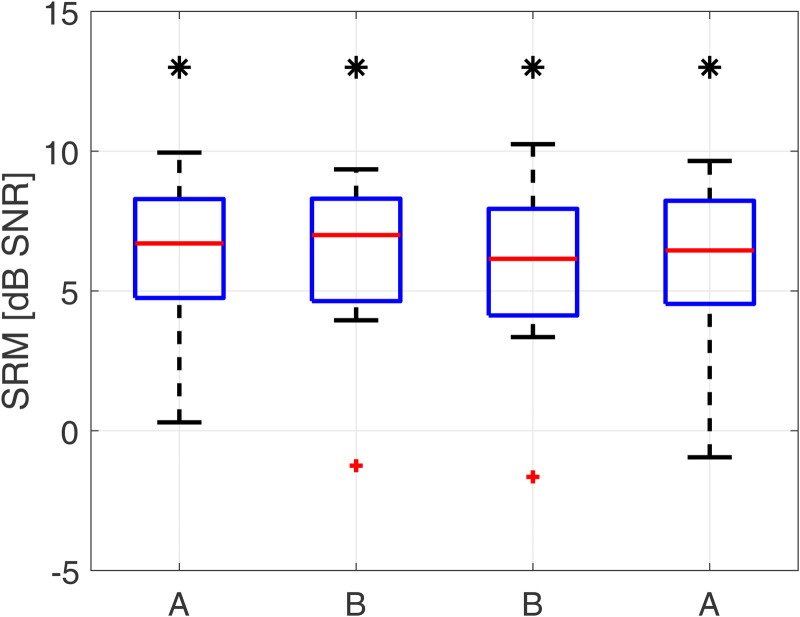
Spatial release from masking (SRM) for all conditions as boxplots (red line: median; box: 1^st^ to 3^rd^ quartile; whiskers: minimum and maximum without outliers; outliers in red). Stars above boxes indicate significant difference from a distribution with a median of 0 dB SNR (*p* < .05).

Figure 7 shows the measured SRM for each delay condition. For each device delay mismatch condition, subjects showed SRM significantly different from zero. Friedman tests showed no significant difference in SRM due to programmed device delay (*χ*^2^(3) = 4.42; *p* = .2). Subject specific SRM as well as SRTs for all conditions can be found in the Supplemental Materials.

## Discussion

This study investigated the effect of compensation of a mismatch in stimulation timing, a so-called device delay mismatch, in bimodal CI/HA users using MED-EL CIs. The primary goal of the study was to investigate, whether longer familiarization periods to changes in device delay mismatch of 3–4 weeks lead to further improvements in sound source localization accuracy than a shorter time span (1 hour) as reported by Angermeier et al. (2021). Furthermore, the effects of a device delay mismatch compensation on speech understanding in noise were investigated. As MED-EL has implemented the possibility to delay the CI stimulation in their latest generation of speech processors via the fitting software, long familiarization periods became feasible.

The ultimate question of this study was, whether this new feature can be readily used by CI fitting specialists without access to a measurement setup to quantify HA delays by using the HA delays provided by the manufacturers and without further alteration to the CI fitting used by the patients. From the current data, several questions concerning the use of device delay mismatch compensation by delaying CI stimulation in bimodal MED-EL CI users can be addressed.

### Sound Localization

The overall results for sound source localization accuracy were in line with previous findings reported by Angermeier et al. (2021). A significant improvement in sound localization accuracy occurred in signed bias when the device delay mismatch was compensated acutely. For the RMS error, the pairwise comparisons did not reveal a significant improvement in this group of subjects after acute device delay mismatch compensation. This can possibly be explained by the already good overall precision without a device delay mismatch compensation in some of the subjects. While previous studies only applied familiarization periods of an hour, the non-significant difference in signed bias and RMS error between the first and second B conditions suggests that any positive effects of device delay mismatch compensation are in fact acute and do not improve further after familiarization of three weeks in our subject group. However, it is unclear if this effect would still be non-significant for longer familiarization periods or a larger sample size. For the signed bias, compensation of the device delay mismatch through delayed CI stimulation led to test results that did not differ significantly from a distribution with an “optimal” signed bias (i.e., 0°) as its median, as Wilcoxon signed-rank tests revealed. As in previous studies, acute reintroduction of a device delay mismatch led to significant deterioration of localization accuracy in terms of signed bias and RMS error. No significant difference between the two conditions without compensation of a device delay mismatch (first and second A condition), could be observed. This is in line with previous studies on the subject. The best median performance in our subject group with an RMS error around 30° was well in line with best localization performance without compensation of the device delay mismatch seen in other studies investigating sound localization in bimodal listeners (Ching et al., 2004; Dunn et al., 2005, 2010; Veugen, Hendrikse, et al., 2016).

By testing with the measured HA delay as well as the HA delay given by the manufacturers acutely in the first B condition, the effects of the selected delay were investigated. RMS errors differed significantly but the absolute difference was rather low with a mean difference of 2.8°. The comparison of signed bias did not reveal significant differences. Combined with the findings that the HA delays measured in the lab and the HA delays provided by the HA manufacturers differed only minimally, this showed that this using manufacturer-provided delays is a feasible way for CI audiologists to compensate the device delay mismatch and improve sound localization performance.

Exploratory linear regression between initial localization accuracy showed no significant correlation with the HA delay and thus with device delay mismatch between CI and HA stimulation in our subjects. This might indicate that bimodal subjects are able to adapt to a device delay mismatch to some extent with the effect possibly being non-linear; however, this effect should be investigated with a bigger sample size since the data show a trend towards a positive correlation. Further evidence for this assumption came from correlating the HA delay with the initial improvement in sound localization accuracy. No correlation between performance improvement after acute device delay mismatch compensation and the magnitude of the device delay mismatch could be observed. These findings might have their roots in a somewhat limited ability to compensate the effects of a device delay mismatch and a lower precision of sound localization in bimodal listeners, with both these boundaries limiting the range of localization accuracy in bimodal subjects. Limited ability of the auditory system to compensate for delay offsets between both ears over the course of several days has been shown by Trapeau and Schönwiesner (2015) in normal-hearing subjects. When a static delay of 625 µs was introduced to one ear via a constantly worn earplug, the mean signed error in sound localization of the subjects acutely deteriorated from 0° to approximately 25° towards the ear provided with the earplug. Interestingly, after one day of familiarization mean signed errors got better by roughly 12°. On the following days of testing, no further improvements due to familiarization were reported. This ceiling effect of plasticity might also be seen in the bimodal subjects in this study and seems not directly connected to the magnitude of the device delay mismatch. Further evidence of a limitation of this plasticity could be seen in the signed bias being significantly different from zero in the initial A condition and localization judgements being biased towards the faster modality. With the improvement in localization accuracy not being correlated with the magnitude of compensated device delay mismatch, this might have its roots in an upper precision limit for sound source localization in bimodal subjects of around 30° RMS error for best performances (Ching et al., 2004; Dunn et al., 2010, 2005; Veugen, Hendrikse, et al., 2016). Only one subject in one study by Seeber et al. (2004) has been reported to localize almost as precisely as normal-hearing listeners but can be considered an outlier in the broader context of the literature. This limited localization precision could be due to the lack of spectral overlap in bimodal subjects, with the CI not stimulating low frequencies sufficiently and the hearing loss on the HA side in the high frequency regions further limiting access to ILD cues and also missing ITD cues, which dominate sound localization in normal-hearing listeners (Seeber et al., 2004; Wightman & Kistler, 1993). However, this limit could also be due to the CI cue encoding alone, limiting sound localization to an RMS error of approximately 30°. This limitation has been shown by Dorman et al. (2016) in bilateral subjects, which do not have a device delay mismatch between their devices and good spectral overlap between both ears. So, it could be argued that this is not an effect only affecting bimodal listeners, but CI users in general and is rooted in the CI signal processing, not sufficiently encoding ITDs. This is further supported by studies showing that SSD CI users with normal acoustic hearing in one ear use ILDs and not ITD for sound localization (Dirks et al., 2019; Dorman et al., 2015).

When comparing the mean performance in sound source localization with and without the device delay mismatch compensation and acute improvement due to device delay mismatch compensation, highly significant correlations could be found for the RMS error and signed bias. This means that in our sample of subjects, bimodal CI/HA users with worse sound source localization accuracy profited more from the compensation of the device delay mismatch. This might also be connected to the upper performance limit in terms of RMS error in bimodal subjects discussed above. When subjects were already close to the limit of achievable localization accuracy this might limit further improvement through device delay mismatch compensation. This is underlined by the best three subjects showing a decrease in localization precision in terms of RMS error. This could mean that these subjects already adapted to their device delay mismatch to reach the best achievable localization performance in bimodal subjects and the change in device delay mismatch can initially lead to a decreased performance since the subjects are still somewhat adapted to their mismatch, thus possibly overcompensating.

When the compensation of the device delay mismatch was removed acutely, a significant correlation between HA delay and thus magnitude of device delay mismatch and decline in sound localization accuracy could be observed. This further supports the hypothesis, that the non-significant correlation between baseline performance and HA delay compensation could have its roots in adaptation to desynchronization between CI and HA. When subjects were used to a compensated device delay mismatch a higher magnitude of device delay mismatch did mean a higher decay in sound localization accuracy. This implies that bimodal subjects can familiarize to changes in device delay mismatch to some extent.

To further understand the familiarization processes to a device delay mismatch studies with shorter time intervals between tests could be conducted with possibly daily testing of sound source localization accuracy over the course of several days as done by Trapeau and Schönwiesner (2015), so as to given sufficient training as to eliminate training bias.

### Speech Tests

SRM was assessed by comparing SRTs between two spatial conditions with speech and noise being collocated at 0° azimuth (S_0_N_0_) and with speech coming from the front and noise coming from the ±90° azimuth on the HA ear (S_0_N_HA_).

For SRM, no significant effect of device delay mismatch was found, whereas this group of subjects did show significant SRM for all tested conditions. The reason for this might be that bimodal subjects cannot utilize binaural cues when it comes to SRM, but rather monaural head shadow cues. This is well in line with previous studies reporting SRM in bimodal HA/CI subjects being only moderated by monaural head shadow and not by binaural unmasking based on ITD (Dieudonné & Francart, 2020; Williges et al., 2019). Further, this is well in line with a previous study investigating the effects of interaural time offsets on SRM when only ILD are present in the signal, showing no effect of a device delay mismatch (Angermeier et al., 2022). Only if sufficient ITD coding and sensitivity can be achieved in bimodal subjects in the future, a device delay mismatch might impair SRM.

## Conclusions

From the presented data, several conclusions can be drawn on the influence of a compensated device delay mismatch in bimodal CI/HA users.

The first conclusion is that the positive effects of a device delay mismatch compensation on sound localization can be achieved with the subjects’ everyday fitting, using manufacturer-provided HA delays without the need to measure HA delays individually. This is most relevant to the broader use of the compensation of device delay mismatch as a fitting parameter in one manufacturer's current CI programming software.

Further, we conclude that the positive effects seen in sound localization accuracy are in fact acute and do not further improve after familiarization of 3–4 weeks.

The magnitude of HA delay shows no direct correlation with sound localization accuracy when the device delay mismatch is present, nor on improvement after compensation of device delay mismatch. This is thought to have its roots in familiarization to the device delay mismatch and an upper performance level in bimodal or even CI sound source localization accuracy, independent of device delay mismatch.

Even with improvements in RMS error being limited by some constraints like the performance limit for sound localization with CI stimulation, the signed bias in bimodal subjects can be reduced to roughly 0°. This means that a compensation of a device delay mismatch can eliminate the systematic shift in localization judgements towards the faster device and effectively re-center the perceived origin locations of sounds.

Without prior familiarization, detrimental effects of a device delay mismatch on sound localization correlated significantly with the magnitude of the device delay mismatch.

SRM was not affected by changes in device delay mismatch, which confirms that bimodal subjects rely on monaural better-ear listening in this task. As soon as future CI sound coding strategies allow for the access to ITD cues for this task, the effect of a device delay mismatch on spatial unmasking should be reinvestigated. A final conclusion that is especially important for bimodal subjects struggling with sound localization is that bimodal subjects with poor localization performance benefit most from the compensation of a device delay mismatch, thus can benefit most from this new fitting parameter.

## Supplemental Material

sj-docx-1-tia-10.1177_23312165231171987 - Supplemental material for Clinical Feasibility and Familiarization Effects of Device Delay Mismatch Compensation in Bimodal CI/HA Users Click here for additional data file. Supplemental material, sj-docx-1-tia-10.1177_23312165231171987 for Clinical Feasibility and Familiarization Effects of Device Delay Mismatch Compensation in Bimodal CI/HA Users by Julian Angermeier, Werner Hemmert and Stefan Zirn in Trends in Hearing

sj-xlsx-2-tia-10.1177_23312165231171987 - Supplemental material for Clinical Feasibility and Familiarization Effects of Device Delay Mismatch Compensation in Bimodal CI/HA Users Click here for additional data file. Supplemental material, sj-xlsx-2-tia-10.1177_23312165231171987 for Clinical Feasibility and Familiarization Effects of Device Delay Mismatch Compensation in Bimodal CI/HA Users by Julian Angermeier, Werner Hemmert and Stefan Zirn in Trends in Hearing
