# Supplementary material for: Clinical Feasibility and Familiarization Effects of Device Delay Mismatch Compensation in Bimodal CI/HA Users
Source: Trends Hear. 2023 May 17;27:23312165231171987. doi: 10.1177/23312165231171987 (PMC10196534; doi:10.1177/23312165231171987)
Supplement: sj-docx-1-tia-10.1177_23312165231171987 - Supplemental material for Clinical Feasibility and Familiarization Effects of Device Delay Mismatch Compensation in Bimodal CI/HA Users [file sj-docx-1-tia-10.1177_23312165231171987.docx]

Supplemental Materials


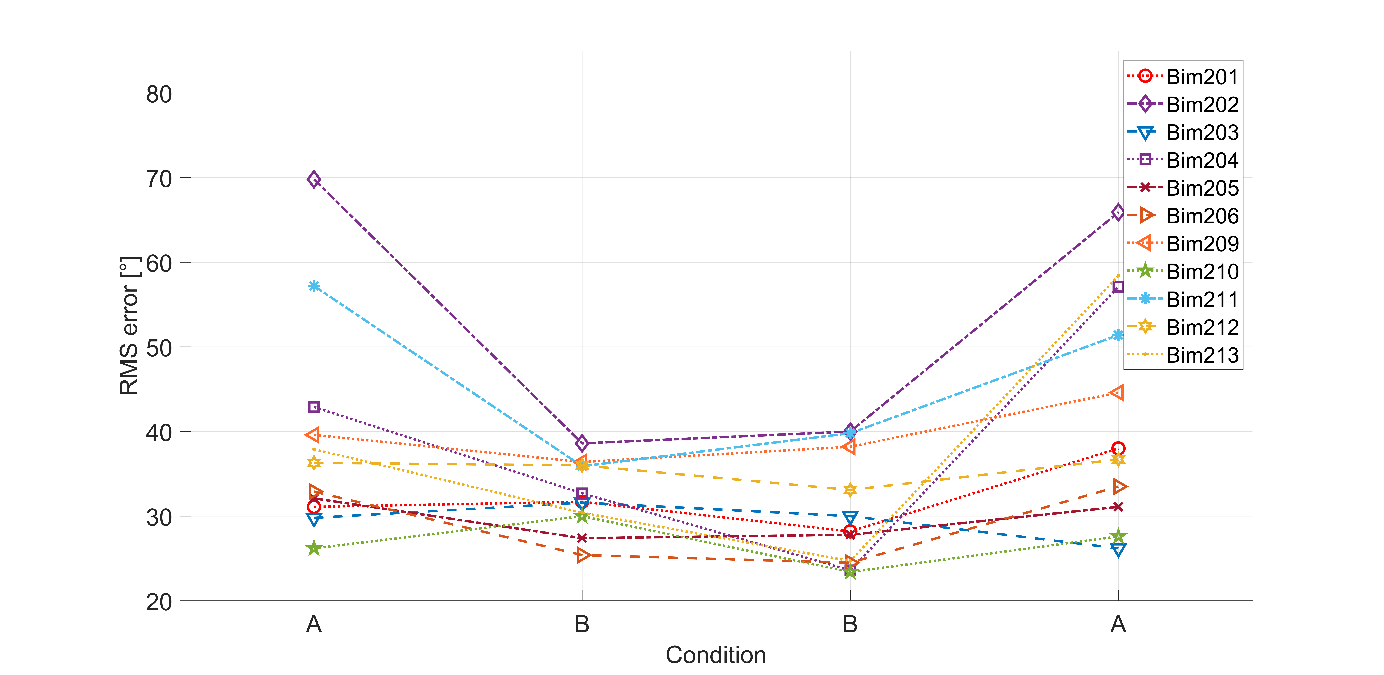


Supplemental figure 1: Subject specific RMS errors between conditions for all 11 subjects


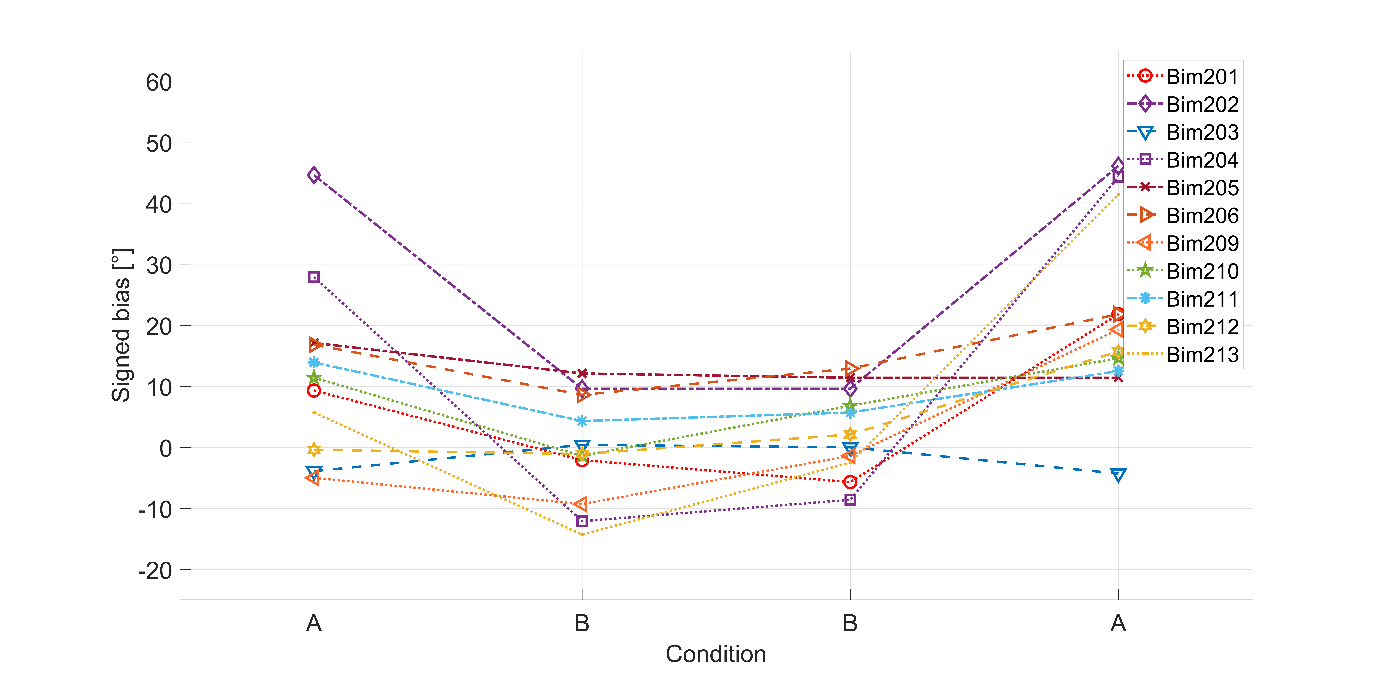


Supplemental figure 2: Subject specific signed bias between conditions for all 11 subjects


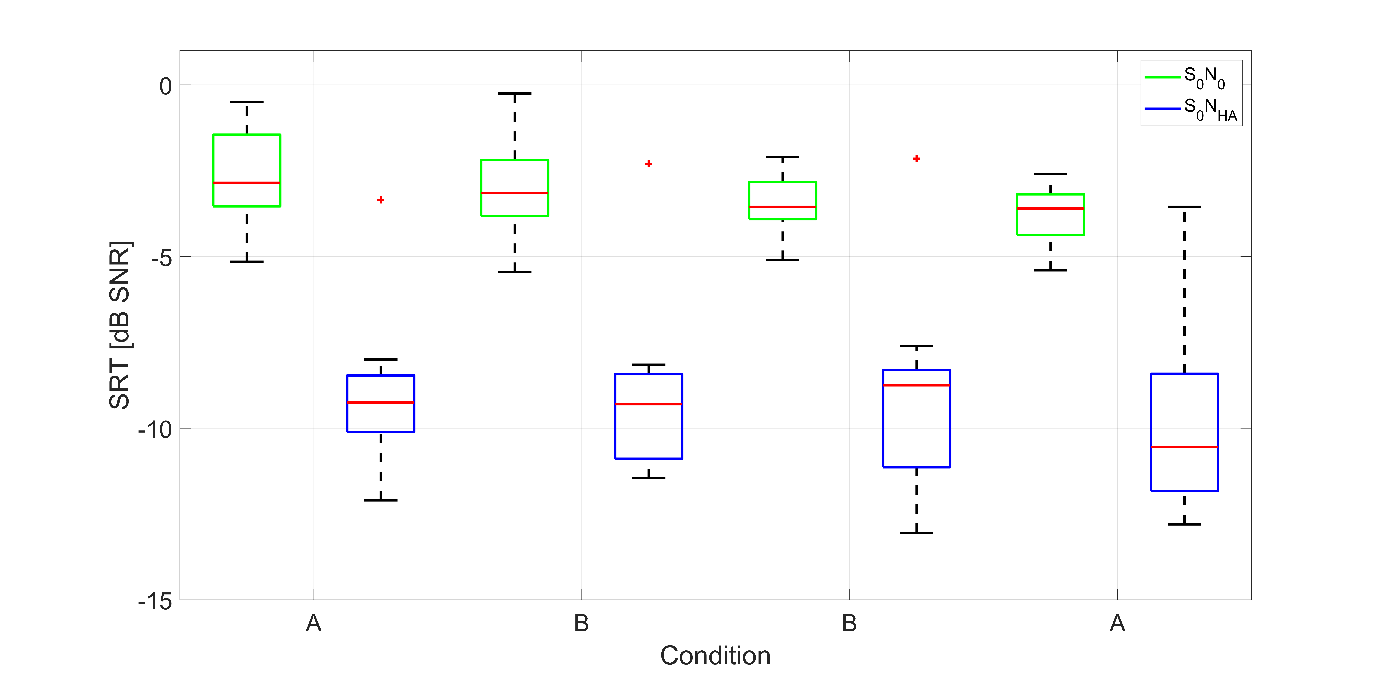


Supplemental figure 3: Speech reception thresholds (SRT) for speech and noise collocated at 0° azimuth (S_0_N_0_) and speech at 0° azimuth and noise from 90° or -90°, dependent on the side the subjects wore their hearing aid (S_0_N_HA_) as boxplots (red line: median; box: 1^st^-3^rd^ quartile; whiskers: minimum and maximum without outliers; outliers in red).


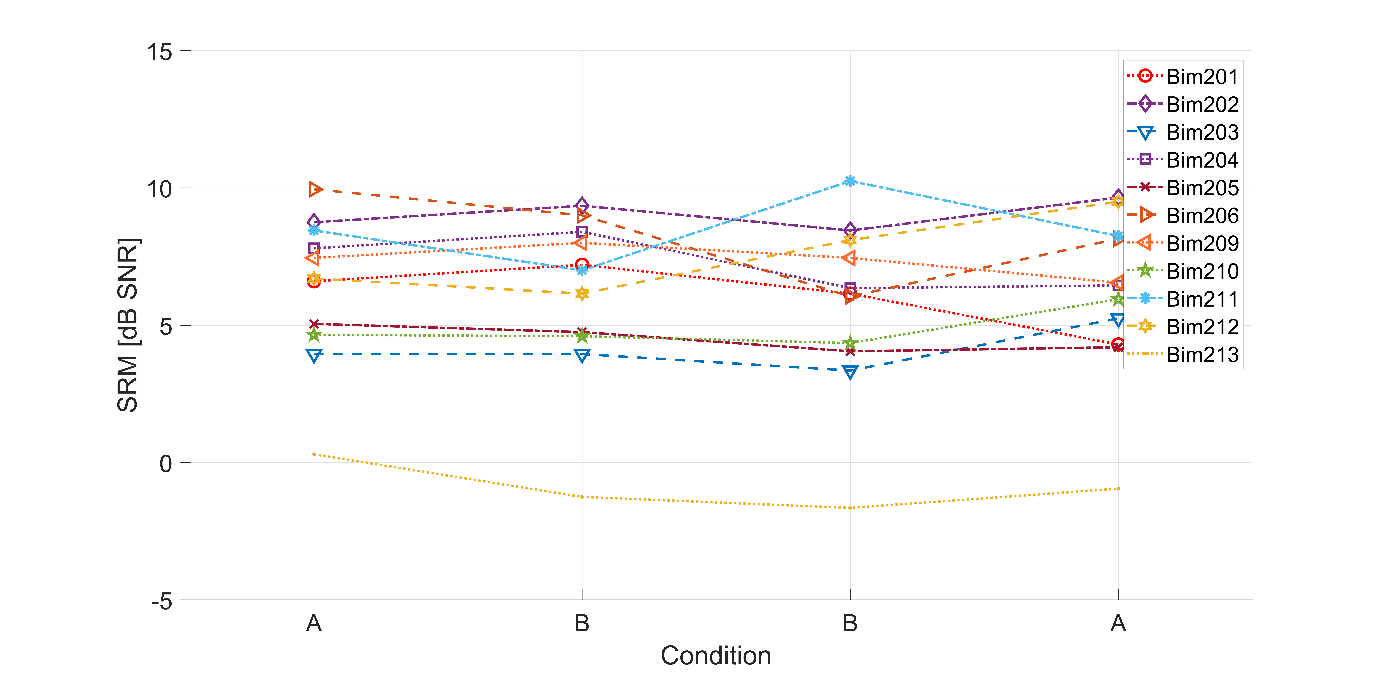


Supplemental figure 4: Subject specific spatial release from masking (SRM) over all conditions for all 11 subjects
